# Positive symptoms of schizophrenia and their relationship with cognitive and emotional executive functions

**DOI:** 10.1186/s41235-022-00428-z

**Published:** 2022-08-12

**Authors:** Pamela Ruiz-Castañeda, Encarnación Santiago Molina, Haney Aguirre Loaiza, María Teresa Daza González

**Affiliations:** 1 Neuropsychological Evaluation and Rehabilitation Center (CERNEP), University of Almeria, Carretera de Sacramento, s / n. La Cañada de San Urbano. 04120, Almeria, Spain; 2 Department of Psychology, University of Almeria Spain, Carretera de Sacramento, s /n. La Cañada de San Urbano. 04120, Almeria, Spain; 3 Mental Health Hospitalization Unit of Torrecárdenas University Hospital, Calle Hermandad de Donantes de Sangre, s/n, 04009 Almería, Spain; 4 Department of Psychology, Catholic University of Pereira, Avenida Sur/Las Americas Cra 21 # 49-95, Pereira, Colombia

**Keywords:** Cognitive executive functions; Socio-emotional executive functions; Schizophrenia, Positive symptoms, Fronto-subcortical syndromes

## Abstract

**Background:**

Positive symptoms of schizophrenia are associated with significant difficulties in daily functioning, and these difficulties have been associated with impaired executive functions (EEFF). However, specific cognitive and socio-emotional executive deficits have not been fully established.

**Objective:**

The present study has several objectives. First, we aimed to examine the specific deficits in cognitive and socio-emotional EEFF in a group of patients with schizophrenia with a predominance of positive symptoms, as well as to determine if these patients present clinically significant scores in any of the three fronto-subcortical behavioral syndromes: Dorsolateral, Orbitofrontal, or Anterior Cingulate.

**Method:**

The sample consisted of 54 patients, 27 with a predominance of positive symptoms, and 27 healthy controls matched for gender, age, and education. The two groups completed four cognitive and three socio-emotional EEFF tasks. In the group of patients, positive symptoms were evaluated using the scale for the Evaluation of Positive Symptoms (SANS), while the behavioral alterations associated with the three fronto-subcortical syndromes were evaluated using the Frontal System Behavior Scale (FrSBe).

**Results:**

The patients, in comparison with a control group, presented specific deficits in cognitive and socio-emotional EEFF. In addition, a high percentage of patients presented clinically significant scores on the three fronto-subcortical syndromes.

**Conclusion:**

The affectation that these patients present, in terms of both cognitive and emotional components, highlights the importance of developing a neuropsychological EEFF intervention that promotes the recovery of the affected cognitive capacities and improves the social and emotional functioning of the affected patients.

## Introduction

The study of the positive symptoms (PS) of schizophrenia (such as prominent delusions, hallucinations, formal thought disorder, and bizarre behavior) is of particular interest both because of the severity of these symptoms and their consequences for the daily functioning of the patient and their impact on their caregivers. This psychotic clinic is usually associated with more significant social stigma and a higher rate of relapses and hospitalizations (Green, 1996; Holmén et al., 2012).

From a neuropsychological point of view, current research has realized that the study of neurocognition has important implications for understanding the prognosis, treatment, and neural systems of schizophrenia (Green et al., 2019; Molina & Tsuang, 2020; Seidman & Mirsky, 2017). Various investigations have suggested that the most pronounced neurocognitive deficits in these patients could occur in executive functions (EEFF) (Addington & Addington, 2000; Díaz-Caneja et al., 2019; Fonseca-Pedrero et al., 2013; Mingrone et al., 2013; Nieuwenstein et al., 2001). These functions are directly related to the quality of life and are considered significant predictors of the patient's prognosis (Bobes García & Saiz Ruiz, 2013). Several studies have highlighted these deficits as a strong predictor for the development of psychiatric disorders (Ancín et al., 2013; Sawada et al., 2017). Thus, the study carried out by Bolt et al. (2019) in patients with “ultra-high risk” of suffering from psychosis found that the EEFF were the only neurocognitive domain that emerged as a significant predictor of the transition to threshold psychosis full. The patients who had more pronounced deficits in this domain were those who developed psychosis in a mean period of 3.4 years. Similarly, Eslami et al. (2011) found that EEFF deficits at baseline were significant predictors of social functioning and occupational decline within one year. Therefore, these types of results could indicate that FFEE deficits may be a highly sensitive indicator of disease transition risk and poor functional outcomes.

Furthermore, in the scientific literature, a distinction has been established between the more cognitive aspects of EEFF, also called “*cool*” components, and the more socio-emotional, or “*hot*” components (Peterson & Welsh, 2014; Prencipe et al., 2011; Welsh & Peterson, 2014).

*Cool* EEFF include those cognitive processes manifested in analytical and non-emotional situations, primarily associated with the dorsolateral regions of the prefrontal cortex (Henri-Bhargava et al., 2018; Kamigaki, 2019). Within these EEFF, we would find at least three central components: (1) the processes of coding/maintenance and updating of information in working memory (WM); (2) inhibitory control; and (3) cognitive flexibility (Miyake & Friedman, 2013; Miyake et al., 2000). In addition, other more complex functions such as planning, abstract reasoning, or problem-solving are developed from these central components. In contrast, *hot* EEFF include those processes involved in contexts that require emotion, motivation, and tension between immediate gratification and long-term rewards (Zelazo & Carlson, 2012; Zelazo & Mller, 2007). Are mediated by the ventromedial and orbitofrontal cortices that support behaviors that require emotional regulation, decision-making in situations of uncertainty, recognition of facial expressions and their emotional content, as well as in the ability to infer the perspective of others, also known as mentalization or theory of mind (ToM) (Welsh & Peterson, 2014; Zimmerman et al., 2016).

Regarding decision-making in situations of uncertainty, it is a complex process that could be defined as the choice of an option among a set of alternatives, considering the possible results of the choices and their consequences on behavior (Kim & Lee, 2012; Xiao et al., 2012). Within this framework, Damasio (1994) postulates his “Somatic Marker” hypothesis to explain the role of emotions in reasoning and decision-making. In this sense, a Somatic Marker is an automatic emotional response that it is produced by the perception of a certain situation, and which in turn evokes past experiences. Specifically, the neural system for the acquisition of Somatic Marker signals is found in the orbitofrontal and ventromedial portion of the prefrontal cortex. Regarding the theory of the mind, authors such as Zimmerman et al. (2016) describe it as an emotional function that refers to the processes responsible for the perception and identification of emotions, such as empathizing with the affective state of another person. Specifically, the neuroanatomical network associated with ToM includes the medial prefrontal region of the prefrontal cortex, the posterior cingulate cortex, the amygdala, the temporoparietal junction, and the temporal sulcus, bilateral superior–posterior (Amodio & Frith, 2006; Ilzarbe et al., 2021; Zemánková et al., 2018).

Regarding the alterations in *cool* EEFF presented by patients with a predominance of PS, the results reported to date are inconclusive. On the one hand, studies that have analyzed EEFF through classical paper-and-pencil neuropsychological tests (e.g., Wisconsin Card Sorting Test; Trail Making Test A and B) have reported poor performance in these patients, suggesting general executive impairment (Addington et al., 1991; Zakzanis, 1998). Moreover, correlations have been reported between PS such as formal thought disorders and persistently bizarre behavior with *cool* executive components, such as inhibition and cognitive flexibility, pointing to a marked deficit in inhibitory control (Brazo et al., 2002; Laplante et al., 1992; Li et al., 2017; Subramaniam et al., 2008). On the other hand, other symptoms such as delusions and hallucinations have been moderately related to difficulties in processing speed, cognitive flexibility, and information updating processes in WM (Ibanez-Casas et al., 2013; Laloyaux et al., 2018). It has even been proposed that the PS are possible consequences of the deficits in self-monitoring capacity that are shown by these patients (Spironelli & Angrilli, 2015).

However, and in contrast to these investigations, other studies suggest conservation of EEFF in these patients (Berenbaum et al., 2008; Clark et al., 2010) or at least a minimal relationship with PS. Thus, some studies report low or null correlations between symptoms such as delusions or hallucinations and performance on verbal fluency, WM, and attention tasks (Berenbaum et al., 2008). Similarly, null correlations have been observed between delusions and hallucinations and performance on tasks that assess resolution problems, working memory, verbal and visual memory, and processing speed, and, using these same tasks, low or moderate correlations with symptoms such as formal thought disorders or bizarre behavior (Ventura et al., 2010).

An important question is whether these results could be influenced by the clinical or socio-demographic variables of the sample. In this regard, some studies (Addington et al., 1991; Zakzanis, 1998) have concluded that performance on EEFF tests is not related to the age of the participants, the number of admissions, the age of disease onset, or type of medication (chlorpromazine equivalents).

The literature on socio-emotional or *hot* EEFF has also yielded mixed results. Regarding decision-making in situations of uncertainty (participants do not have direct information about the disadvantages of their choices and do not have the opportunity to establish a reasonable strategy at the beginning of the task (Pedersen et al., 2017)), the studies that have examined the performance of patients with a predominance of PS in the Iowa Gambling Task (IGT) show inconsistent results. Some studies have found negative correlations between symptoms such as hallucinations and prominent delusions and performance on this task compared to controls. In particular, a higher PS score was correlated with a lower Net Score (number of disadvantageous options minus the number of advantageous options), fewer advantageous choices (Struglia et al., 2011), and a greater number of disadvantageous choices (Pedersen et al., 2017). Other studies, however, using the same paradigm (IGT), did not find differences in performance compared to controls or correlations between IGT performance and symptomatology (Evans et al., 2005; Ritter et al., 2004; Wilder et al., 1998).

Regarding the ability to infer mental states or theory of mind, a generalized deterioration has been reported in these patients, particularly in those with marked PS such as delusions and hallucinations (Corcoran et al., 1995). However, in contrast, it has been hypothesized that for the development of certain PS such as persecutory delusions, an intact theory of mind is required, since this is necessary for inferring the intentions of others, even though these inferences are not correct (Peyroux et al., 2019; Walston et al., 2000).

When analyzing the possible influence of clinical and demographic variables on the results of these studies, although the studies have not considered this as a primary objective, the patients were matched with the control group in terms of age, gender, or education, which has led the authors to suggest that these variables are not the cause of the results and that patients perform the task in a different way to controls (Corcoran et al., 1995; Peyroux et al., 2019).

On the other hand, from a neuropsychological point of view, it has been suggested that the heterogeneity and diversity of symptoms shown by patients with schizophrenia could be a consequence of a malfunction of brain circuits of fronto-subcortical origin (Fornito et al., 2012; Penadés & Gastó, 2010). According to this approach, schizophrenia tends to be considered as a neuronal connectivity disorder and its different symptomatology could be explained by using the distributed neural network model (Goldman-Rakic, 1994; Pantelis & Brewer, 1995; Wang et al., 2014). This model posits that control of any cognitive function is distributed across several interconnected nuclei throughout the brain. The interruption of any of these nuclei or their interconnections would produce changes in cognitive function (Baars & Cage, 2010). In this sense, the involvement of these prefrontal areas and/or their connections with other subcortical regions (e.g., the fronto-subcortical circuits of prefrontal origin: Dorsolateral syndrome, related to executive deficits; Orbitofrontal syndrome, related to disinhibition; and syndrome Anterior Cingulate, related to apathetic behaviors (Bonelli & Cummings, 2007; Tekin & Cummings, 2002)), could result in specific deficits in the different *cool* and *hot* components of the EEFF (Slachevsky Ch. et al., 2005).

In this sense, and regarding the brain areas involved in the PS of schizophrenia, these are not yet fully established. Some inferences in this regard have been obtained from patients with traumatic brain injury (TBI) who have developed clinical symptoms and behaviors like those presented in patients with PS in schizophrenia after the injury. Psychotic symptoms such as hallucinations, persecutory delusions, and thought disorders (loosening of associations, tangentiality, or thought blockage) occur more frequently in patients with TBI than in the general population (Fujii & Ahmed, 2002; Sachdev et al., 2001).

Similarly, a high percentage of patients with TBI also show significant alterations upon neuropsychological examination, similar to those presented by patients with psychotic symptoms, particularly in executive functions and memory (Berrios, 2013). These alterations have been associated with post-traumatic structural lesions located in different brain regions, such as the frontal cortex (dorsolateral and orbitofrontal), and, in those structures that form the so-called fronto-subcortical circuits (Alexander et al., 1986; Pettersson-Yeo et al., 2011).

Therefore, and in summary of the above, two main conclusions can be drawn. First, a review of the current literature has revealed inconclusive results regarding the level of alteration in *cool* and *hot* EEFF presented by schizophrenic patients with a predominance of PS. Moreover, there is no conclusive relationship between specific executive components and PS.

Second, the findings of neuroanatomical studies on the affectation of the fronto-subcortical circuits in TBI patients who develop behaviors and PS similar to those presented by patients with schizophrenia could suggest possible alterations of these circuits in schizophrenic patients. Therefore, it is possible that patients with schizophrenia with a predominance of PS present behaviors associated with the so-called fronto-subcortical syndromes (Dorsolateral Prefrontal Syndrome, related to executive deficits; Orbitofrontal syndrome, related to disinhibition; and Anterior or Mesial Cingulate Syndrome, related to apathic behaviors). However, to our knowledge, there is no previous study that has explored the possible involvement of the fronto-subcortical circuits in patients with positive symptoms from the presence of behaviors associated with fronto-subcortical syndromes.

Thus, the present study had several objectives. First, we aimed to study the specific deficits in *cool* and *hot* EEFFEF in a group of patients with schizophrenia with a predominance of PS, in comparison with a control group of healthy participants matched for age, gender, and educational level. Second, we set out to study the influence of the main clinical variables (years of evolution of the disease, clinical treatment device, and pharmacological treatment) on executive task performance shown by these patients. Third, we aimed to explore the possible relationship between the severity of PS (hallucinations, delusions, bizarre behavior, and formal thought disorders) with performance on both *cool* and *hot* EEFF tasks. And, finally, we wanted to confirm if these patients present clinically significant scores on any of the three fronto-subcortical behavioral syndromes: Dorsolateral, Orbitofrontal, or Anterior Cingulate. (These were measured through the self-reported version of the Frontal System Behavior Scale—FrSBe.)

Considering the previous literature concerning our first objective, we expect psychotic patients with a predominance of PS to show significantly poorer performance on the EEFFEF tasks in comparison with healthy controls. Moreover, in terms of clinical variables, we expect that the years of disease duration, the clinical treatment device, and the type of pharmacological treatment could affect the performance of patients on EEFF tasks.

Regarding the third objective, we expect that the patients with the highest scores on the scale for the Evaluation of Positive Symptoms (SAPS) also show poorer performance on the EEFFEF tasks. Regarding the fourth objective, we anticipate that these patients with a predominance of PS will present some of the frontal behavioral syndromes.

## Materials and methods

### Participants

**Fig. 1:**
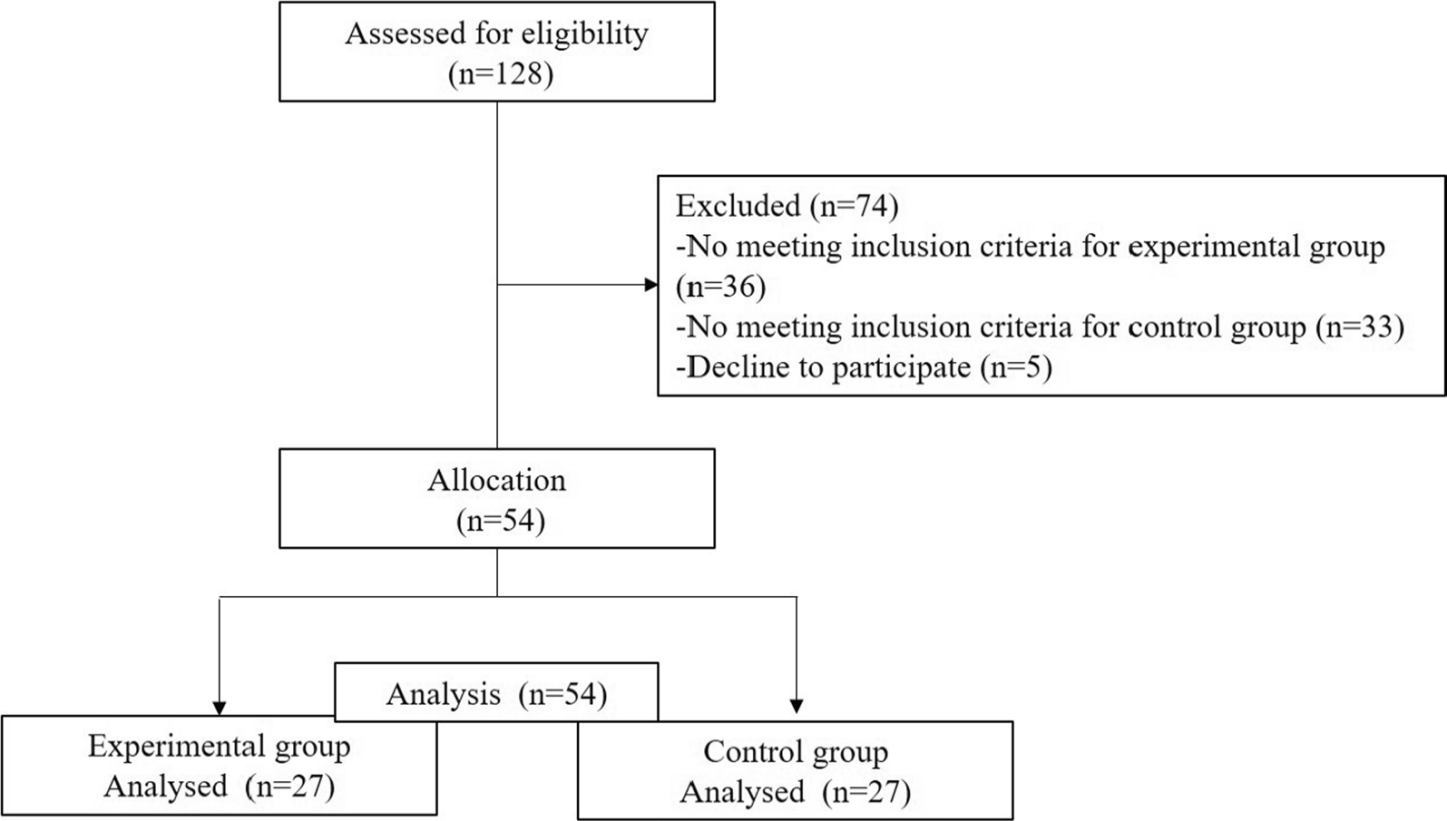
Flow of participants throughout the study

The initial sample consisted of 128 participants (age range: min = 20, max = 61, *M*_age_ = 37.4, *SD* = 10.7). The selection process is shown in Fig. 1. The final sample consisted of *n* = 54 participants (age range: min = 20, max = 60), of both genders: men (*n* = 49, 74.2%, *M*_age_ = 43.6, *SD* = 11.0), women (*n* = 17, 25.8%, *M*_age_ = 44.2, *SD* = 11.0); 27 patients with schizophrenia, and 27 participants assigned to the control group.

### Criteria for inclusion and exclusion of the experimental group

#### Inclusion criteria

1. Patients between 18 and 57 years.
2. Defined diagnosis of schizophrenia
3. Minimum of two years of evolution of the disease
4. PS predominance. For this, those patients who showed a higher percentage score in the Evaluation of Positive Symptoms (SAPS) than in the Scale for the Evaluation of Negative Symptoms (SANS) were selected.
5. Likewise, the psychopathological stability and motivation of the patient were considered, selecting psychopathologically stable patients to carry out the evaluation. The referral psychiatrist established this criterion based on prior knowledge of the patient's clinical status, ensuring sufficient compensation and motivation for participation in the study.

#### Exclusion criteria

1. Participants whose main diagnosis is an organic mental disorder, a different medical or psychological illness.
2. Electroconvulsive treatment in the last 2 years,
3. Patients with very low motivation for active participation in the study.

### Criteria for inclusion and exclusion of the control group

#### Inclusion criteria

1. Subjects between 18 and 57 years
2. Subjects who could be matched with the patients in age, gender, and educational level.
3. Have no history of mental, neurological, or substance abuse illness,
4. Not be medicated with any psychotropic medication.

#### Exclusion criteria

1. Those participants who did not meet the inclusion criteria were excluded.

The patients were selected from the various medical facilities of the Mental Health unit of the reference Hospital Complex of the city. Regarding the socio-demographic variables, three levels were established according to the years of schooling: basic (6 years), medium (between 7 and 12 years), and high (more than 12 years). Regarding the clinical variables, for the duration of the illness, two levels were established according to the sample mean: a group with a shorter duration off illness (less than 11 years) and another group with a longer duration of illness (more than 11 years). Regarding clinical treatment service, two levels were established according to whether they received treatment in an inpatient or outpatient setting. For pharmacological treatment, two levels were established according to whether they took typical or/and atypical medications, and other medications unrelated to mental illness. The control group was matched with the patients in terms of age, gender, and years of schooling. The selected participants had no history of mental, neurological, or substance abuse illness and were not taking any psychotropic medications. The study was carried out in accordance with the guidelines of the Declaration of Helsinki and was approved by the Research Ethics Committee of Centro-Almería belonging to the Torrecárdenas Hospital Complex in the city of Almería (protocol code 52,780. approval date: 26 / 10/2014). The patients/participants provided their written informed consent to participate in this study.

## Assessment

### Execution tasks

For the study of *cool* EEFF, four different neuropsychological tasks were used: 1) the Sternberg-type task, which assesses the processes of encoding/maintaining information in working memory (WM); 2) the 2-back task, which evaluates the monitoring and updating processes of information in WM; 3) the Number–Letter task, which assesses cognitive flexibility or ability to change or alternate the mental set; and 4) a computerized version of the Tower of Hanoi (THO), which evaluates the planning processes involved in the preparation of ordered sequences of actions to achieve specific objectives.

Regarding the *hot* EEFF, the following three tasks were used: 1) a computerized version of the Iowa Gambling Task (IGT) which assesses decision-making processes in situations of uncertainty; 2) a computerized task for the recognition of facial emotional expressions, and 3) a pencil and paper version of the Hinting task that evaluates the theory of mind (ToM) (See Table 1). For a more detailed description of the *cool* and *hot* EEFF tasks used in the present study, see Ruiz-Castañeda et al. (Ruiz-Castañeda et al., 2020).

**Table 1:** Tasks to evaluate the components of the *cool* and *hot* executive functions and behavioral scales used in the study

Measure	Instrument
***Cool* components of the EEFF**	
Encoding/maintaining the information in WM	Sternberg-type task(Sternberg, 1966)
Monitoring and updating information in the WM	2-Back Task (Fletcher, 2001)
Ability to change or alternate the mental set	Number–Letter task (Rogers & Monsell, 1995)
Planning	Computerized version of the Tower of Hanoi (Borys et al., 1982)
***Hot* components of the EEFF**	
Decision-making under uncertainty	Computerized version of the Iowa Gambling Task (Bechara et al., 1994)
Facial emotional expression	Facial emotional expression recognition task (Baron-Cohen et al., 1997)
Theory of mind	Spanish version of the Hinting Task (Gil et al., 2012)
**Psyc*hot*ic symptoms**	
Negative symptoms	Scale for the assessment of negative symptoms (Andreasen, 1984)
Positive symptoms	Scale for the Assessment of positive Symptoms (Andreasen, 1984)
**Frontal**-**subcortical syndrome**	
Behavioral disorders of the Frontal systems	Spanish version of the Frontal Systems Behavior Scale (Pedrero et al., 2009)

EEFF: executive function; WM: working memory.

### Scales for the evaluation of psychotic symptoms and frontal behavioral syndromes

To evaluate positive and negative symptoms, the Scale for the Evaluation of Positive Symptoms (SAPS) (Andreasen, 1984) and the Scale for the Evaluation of Negative Symptoms (SANS) (Beck & Chaudhari, 1976) were used. The behavioral alterations associated with the three frontal syndromes: Dorsolateral Syndrome (executive dysfunction); Orbitofrontal Syndrome (disinhibition); and Anterior or Mesial Cingulate Syndrome (apathy), were evaluated using the Spanish version of the Frontal System Behavior Scale (FrSBe) (Grace & Malloy, 2001; Pedrero-Pérez et al., 2009)**.**

### Procedure

For all participants (experimental and controls), the EEFF tasks were administered by two researchers so that one of them always carried out the evaluation, while the second investigator supervised these evaluations. For the patients, the evaluation took place across two individual sessions of approximately 50 min, each with the necessary breaks required by the participant. In the case of the control group, most of them required a single session of approximately 60 min, with the necessary breaks. The evaluation sessions were carried out individually in a quiet room using a laptop.

In the case of patients, the SANS and SAPS scales were administered by the referral physicians (psychiatrists or clinical psychologists). The self-reported version of the FrSBe Scale could be completed by the patient independently (in the researcher's presence) or by the researcher, always trying to ensure the maximum understanding of the questions.

To select psychotic patients with a predominance of PS, the following procedure was applied. Once the patients' referral psychiatrists or clinical psychologists completed the SAPS and SANS scales for each patient, the total scores for each scale were calculated. Each score was then transformed into a percentage. For the SAPS scale, the percentage is calculated based on the maximum score obtained on this scale (170), following the same procedure for the SANS scale (maximum score = 150). Finally, those patients who had a higher percentage on the SAPS scale (*M* = 24.0, *DT* = 16,3) than on the SANS (*M* = 15.1, *SD* = 14.4) were selected.

### Statistical analysis

The data were processed through a descriptive and frequency analysis to characterize the socio-demographic and clinical variables. In the exploratory analysis of the data of the response variables, missing data were found, which were imputed to the median value of each group. Outliers were maintained to ensure consistency with the performance of the evaluated. Gender was matched in each group (n = 17 male, n = 10 female). Age was compared with the Mann–Whitney U test, and education level was assessed with *X*.^*2*^

The direct scores of the neuropsychological tasks were transformed into Z scores. Two multivariate analysis models (MANOVA) were carried out, one with all the measures of the *cool* EEFF tasks and the other with the measures of the *hot* EEFF tasks. The first model was EEFF-*cool* * groups (9 × 2), and the second model was EEFF-*hot* * groups (6 × 2). Assumptions of normality for hypothesis testing were checked through standardized residuals in both groups. The assumption of equality of covariances was estimated with Box's test, and the multivariate Lambda test of Wilks *(Λ)* was used. The analysis of multiple comparisons between patients and controls was corrected with Sidak’s procedure. For the comparisons that showed significant differences, the confidence interval (95% CI) of the differences was reported. The effect size was estimated with eta squared (*η*_*p*_^*2*^), using the following values: < 0.01 small, 0.06 moderate, and > 0.14 strong (Cohen, 1988).

Pearson's r correlation analyses were conducted between PS and EEFF tasks. To check whether the patients with PS had clinically significant scores in any of the three frontal behavioral syndromes, the direct scores obtained on the FrSBe scale were converted into standardized scores (*T*) according to the age, education, and gender of the participant. With these T scores, three ranges of affectation can be obtained according to their cutoff point: no risk (< 59 points); high risk or borderline (60 to 64); and clinically significant (> 65). The data analyses were conducted using SPSS v.23.0. Post hoc statistical power (*1-β*) was calculated with G * Power software (Faul et al., 2009).

## Results

No significant differences were found between patients and controls in age [U(*N*_patients_ = 33, *N*_controls_) = 542.0, *z* = − 0.03, *p* = 0.974], gender [*X*^*2*^*(1)* = 0.79*, p* = 0.778], or years of education [*X*^*2*^*(2)* = 0.83*, p* = 0.959]. The socio-demographic and clinical characteristics are shown in Table 2.

**Table 2:** Clinical and socio-demographic variables of the patients and the control group

Variables	Patients *n* = 27	Controls *n* = 27	All *n* = 54
	*f* (%)	*f* (%)	*f* (%)
**Socio-demographic**			
Age_*years old*_ M( ±)	36.4 ± 11.0	38.5 ± 10.5	37.4 ± 10.7
Gender			
Male	17(36.0)	17(36.0)	49(74.2)
Female	10(37.0)	10(37.0)	17(25.8)
Schooling_(years)_			
Basic (< 6)	2(7.4)	2(7.4)	33(50.0)
Medium (7 and 12)	14(51.9)	16(59.3)	19(28.8)
High (> 12)	11(40.7)	9(33.3)	14(21.1)
**Clinical**			
Years of evolution of the disease			
Short	15(55.6)	–	–
Long	12(44.4)	–	–
Clinical treatment device			
In-hospital	10(63.0)	–	–
Outpatient	17(37.0)	–	–
Pharmacological treatment			
Typical/Atypical antipsychotics	23(85,2)		
Other medications	4(14.8)		

a = short (< 11 years), long (> 11 years).

### Cool EEFF tasks

The descriptive data of the *cool* EEFF comparing patients with controls are shown in Table 3. The MANOVA analysis revealed a significant interaction between the *cool* EEFF and the groups [*Wilks’ Λ* = 0.498, *F*(9, 44) = 4.93, *p* < 0.001, *ηp*^*2*^ = *0.50, 1-β* = *0.99*]. Better performance on the *cool* EEFF tasks was observed in the control group.

**Table 3:** *Cool* EEFF. Descriptive statistics. *M(SD)* of transformed score *Z* and multivariate (MANOVA) results for patients and controls

*Cool* EEFF	Direct score		Score Z		*MS*	*F*_(*df*, 1)_	*p*	*ηp*^*2*^
	Patients (*n* = 27)	Control (*n* = 27)	Patients	Control				
**Sternberg-type task**								
Low load_(% Errors)_	13.41(11.8)	4.72(16.7)	0.29(0.7)	− 0.29(1.1)	4.53	4.86	0.032	0.086
High load_(% Errors)_	24.07(11.7)	13.46(15.3)	0.36(0.8)	− 0.36(1.0)	7.21	8.19	0.006	0.136
**2-back task**								
a-prime index_(accuracy)_	0.74(0.2)	0.92(0.1)	− 0.48(1.2)	0.48(0.2)	12.88	16.69	< 0.001	0.243
**Number–Letter task**								
TSC_RT(sec)_	2.0(3.0)	0.6(0.4)	0.30(1.3)	− 0.30(0.2)	4.97	5.38	0.024	0.094
TSC_(errors)_	2.83(10.7)	− 0.61(9.5)	0.17(1.3)	− 0.17(0.1)	1.55	1.57	0.215	0.029
**Tower of Hanoi**								
Short_(Errors)_	0.27(0.3)	0.17(0.3)	0.17(1.1)	− 0.17(0.8)	1.64	1.66	0.202	0.031
Long_(Errors)_	1.71(1.3)	1.55(1.2)	0.06(1.0)	− 0.06(0.9)	0.22	0.22	0.637	0.004
Short_(Latency, sec)_	28.0(11.7)	20.6(11.9)	0.30(0.9)	− 0.30(0.9)	4.87	5.27	0.026	0.092
Long_(Latency, sec)_	67.0(33.8)	57.8(36.2)	0.13(0.9)	− 0.13(1.0)	0.93	0.93	0.339	0.018

TCS = task-switching costs, RT = response time, MS = mean square

A main effect was found in the two conditions of the information *coding/maintenance task in WM* (Sternberg-type task) [low load: *F*(1, 52) = 4.86, *p* = 0.032, *ηp*^*2*^ = *0.08, 1-β* = *0.58*; and high load: *F*(1, 52) = 8.19, *p* = 0.006, *ηp*^*2*^ = *0.136, 1-β* = *0.8*0]. Likewise, a main effect was found for the task of *updating the information in WM* (2-Back task) [*F*(1, 52) = 16.69, *p* < 0.001, *ηp*^*2*^ = *0.243, 1-β* = *0.9*8].

**Fig. 2:**
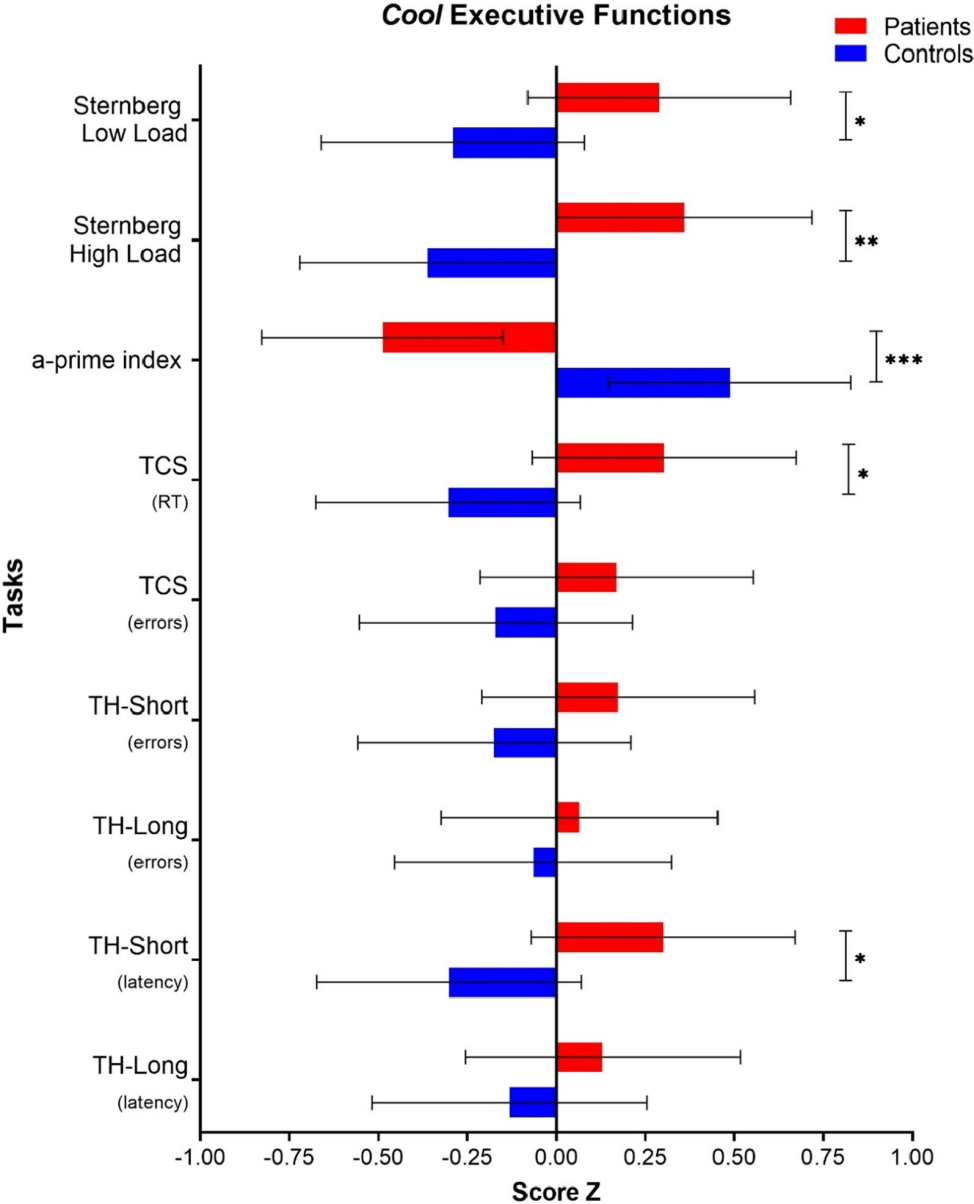
*Cool* EEFF compared between patients and controls. *Note*: TSC = Task-switching costs. RT = Response time. *p < 0.05. **p < 0.01. ***p < 0.001

Regarding performance on the task that assesses *cognitive flexibility* (Number–Letter task), only significant “task-switching costs” (TSC) were observed with reaction time (TSC^TR^) [*F*(1, 52) = 5.38, *p* = 0.024, *ηp*^*2*^ = *0.094, 1-β* = *0.6*24]. Regarding the *planning* task (Tower of Hanoi), only one main effect was observed with the latency measure in the short planning condition [*F*(1, 52) = 5.27, *p* = 0.026, *ηp*^*2*^ = *0.092, 1-β* = *0.6*15] (See Fig. 2).

### Hot EEFF tasks

The descriptive data of the *hot* EEFF comparing patients and controls are shown in Table 4. The MANOVA analysis revealed a significant interaction between the *hot* EEFF tasks and the groups [*Wilks’ Λ* = 0.475, *F*(6, 47) = 8.642, *p* < 0.001, *ηp*^*2*^ = *0.52, 1-β* = *1.0*]. Better performance on the *hot* EF tasks was observed in the control group.

**Table 4:** *Hot* EEFF. Descriptive *M(SD)* of transformed score *Z* and multivariate (MANOVA) results for patients and controls

*Hot* EEFF	Direct score		Score Z		*MS*	*F* (*df*, 1)	*p*	*ηp*^*2*^
	Patients (*n* = 27)	Control (*n* = 27)	Patients	Control				
**Iowa Gambling Task**								
Net Score	0.72(2.4)	1.12(4.0)	− 0.06(0.7)	0.06(1.2)	0.20	0.19	0.65	0.004
**Facial emotional expression**								
Basic emotions_(%Errors)_	17.19(13.5)	10.04(7.0)	0.32(1.2)	− 0.32(0.6)	5.47	5.99	0.018	0.103
Complex emotions_(%Errors)_	5.2(2.7)	2.7(0.8)	0.39(1.0)	− 0.39(0.8)	15.35	21.20	< 0.001	0.290
Basic emotions _RT(sec)_	35.44(10.5)	27.37(8.8)	0.53(1.1)	− 0.53(0.3)	8.07	9.34	0.004	0.152
Complex emotions_RT(sec)_	6.0(3.2)	3.2(1.1)	0.48(1.2)	− 0.48(0.3)	12.67	16.34	< 0.001	0.239
**Hinting task**	13.83(4.4)	18.59(1.4)	− 0.59(1.1)	0.59(0.3)	19.00	29.06	< 0.001	0.359

CI for difference. RT = response time. MS = mean square

Regarding the task that assesses *decision-making under conditions of uncertainty* (Iowa Gambling Task), the analysis of the Net Score measure (Nº of Advantageous choices—Total Nº of disadvantageous choices) did not show a significant effect [*F*(1, 52) = 0.19, *p* = 0.657, *ηp*^*2*^ = *0.004, 1-β* = *0.07*].

**Fig. 3:**
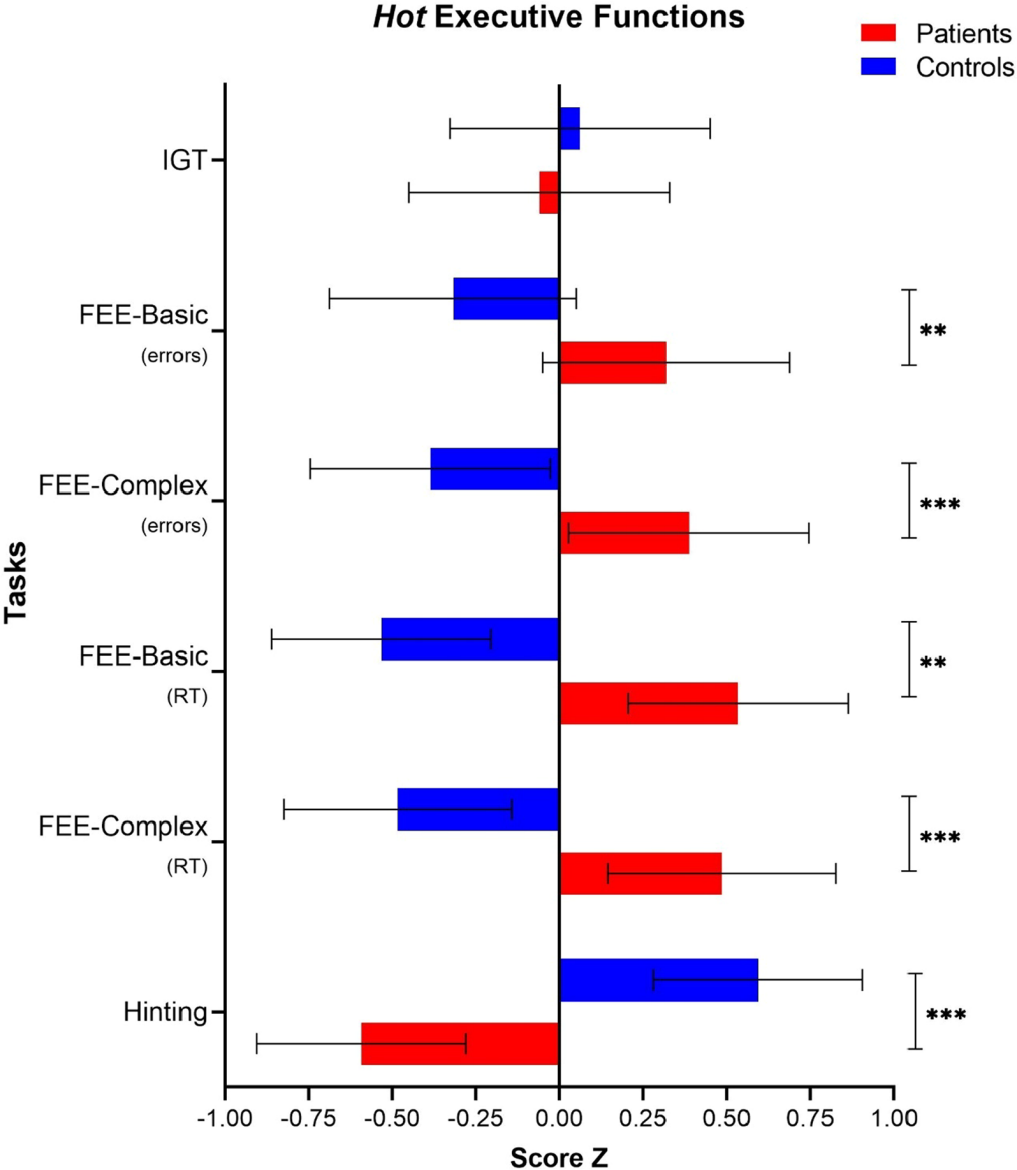
*Hot* EEFF compared between patients and controls. *Note*: IGT = Iowa Gambling Task. FEE =  Facial emotional expressions. RT = Response time. *p < 0.05. **p < 0.01. ***p < 0.001

In contrast, the task that measures the *recognition of facial emotional expressions* showed significant effects on errors, both in basic facial expressions [*F*(1, 52) = 5.993, *p* = 0.018, *ηp*^*2*^ = *0.10, 1-β* = *0.67*], as in complex facial expressions [*F*(1, 52) = 9.34, *p* = 0.004, *ηp*^*2*^ = *0.15, 1-β* = *0.85*]. Similarly, significant effects were also observed in reaction times, both for the condition of basic facial expressions [*F*(1, 52) = 21.20, *p* < 0.001, *ηp*^*2*^ = *0.29, 1-β* = *0.99*], as complex [*F*(1, 52) = 16.34, *p* < 0.001, *ηp*^*2*^ = *0.23, 1-β* = *0.98*]. Finally, the performance of the task that assesses the *theory of mind* (Hinting Task) was significant [*F*(1, 52) = 29.06, *p* < 0.001, *ηp*^*2*^ = *0.35, 1-β* = *1.0*] (See Fig. 3).

### Clinical variables and patient performance in hot and cool EEFF tasks

Regarding the variable *years of disease evolution*, differences were only observed in the errors of the planning task (Tower of Hanoi) in the condition of precision in short planning [*t*(31) =  − 2.51, *p* = 0.034, *d* = 0.71 95%CI (− 1.86, − 0.08)]; that is, patients with a short disease evolution (less than 11 years) showed better performance [*n* = 15; *M* = − 0.25, *SD* = 0.62], than the patients with long disease evolution (more than 11 years) [*n* = 12; *M* = 0.71, *SD* = 1.3]. Based on these results, we wanted to analyze whether the short evolution group showed similar performance to the control group [*n* = 27; *M* = − 0.17, *SD* = 0.88] and found that these two groups did not differ.

Regarding the *clinical device* in which the patients received the intervention, no significant differences were found in performance between patients with an *outpatient intervention* (n = 17) and patients with *in-hospital intervention* (n = 10).

Regarding *pharmacological treatment*, no significant differences were found between the group of patients taking *typical and/or atypical antipsychotics* (n = 23) and those receiving other medication *unrelated to mental illness* (n = 4). Given these results, we wanted to check whether there were significant differences between those patients who were taking typical medications or a combination of typical and atypical (*n* = 4), and those who were only taking atypical medications or other non-psychotropic medications (*n* = 23), finding no significant differences between these two groups.

### Correlations between positive symptoms and performance on the cool and hot EEFF tasks

The results of the correlation analysis between the severity of the PS and performance on the *cool* and *hot* EEFF tasks are shown in Table 5. Regarding the *cool* EEFF tasks, both the *hallucination* symptoms (*r* = − 0.47, *p* = 0.012) and *delusions* (*r* = − 0.39, *p* = 0.044) were related to the planning task (the Tower of Hanoi), in the latency condition in short planning.

**Table 5:** Correlations coefficients (*r*) between positive symptoms and EEFF tasks

	Positive symptoms			
	1	2	3	4
***Cool* EEFF**				
Sternberg-type task				
Low load_(% Errors)_	− 0.00	0.30	0.42^*^	0.13
High load_(% Errors)_	− 0.20	0.15	0.21	27
2-Back Task				
a-prime index_(accuracy)_	− 0.05	0.28	0.03	− 08
Number–Letter task				
TSC_RT(sec)_	− 0.21	− 0.23	0.23	0.44^*^
TSC_(errors)_	0.12	0.00	− 0.12	0.03
Planning				
Short_(Errors)_	− 0.23	− 0.32	− 0.14	− 0.03
Long_(Errors)_	0.11	− 0.17	17	0.19
Short_(Latency. sec)_	− 0.47^*^	− 0.39^*^	− 0.03	0.20
Long_(Latency. sec)_	− 0.31	− 0.17	0.23	0.38^*^
***Hot* EEFF**				
Iowa Gambling Task				
Net Score	− 0.06	0.18	0.03	0.06
Facial emotional expression				
Basic emotions_(%Errors)_	0.18	0.29	0.25	0.17
Complex emotions_(%Errors)_	− 0.04	0.21	0.19	0.24
Basic emotions _RT(sec)_	0.04	0.07	− 0.07	0.17
Complex emotions_RT(sec)_	0.03	− 0.02	− 0.15	0.22
**Hinting task**	0.05	0.01	− 0.09	− 0.46^*^

1= Hallucinations. 2=Delusional ideas. 3= Bizarre behavior. 4=Formal thought disorders. TSC = Task-switching costs. *p < 0.05

Regarding the symptoms of *bizarre behavior*, these correlated with the task of coding/maintaining the information in WM (Sternberg-type task) in the low load condition (*r* = 0.42, *p* = 0.027). *Formal thought disorder* symptoms correlated with the cognitive flexibility task (Number–Letter task) in the TSC^TR^ condition (*r* = 0.44, *p* = 0.022), as well as the reaction times in the long planning condition (*r* = 0.38, *p* = 0.047).

Regarding the *hot* EEFF, the symptoms of *formal thought disorder* correlated with performance on the theory of mind task (Hinting Task) (*r* = − 0.46, *p* = 0.016).

### Frontal Behavioral syndromes in patients with positive symptoms

Regarding the presence of the three frontal behavioral syndromes in patients with PS, we found that for *Dorsolateral syndrome* (executive dysfunction subscale), 81.5% presented a clinically significant score. For *Orbitofrontal syndrome* (Disinhibition subscale), 59.3% had a clinically significant score, while 77.8% had a clinically significant score for the anterior *cingulate syndrome* (Apathy subscale).

## Discussion

The objectives of this work were to (1) study the specific deficits in the *cool* and *hot* EEFF in a group of patients with schizophrenia with a predominance of PS, compared to a control group of healthy subjects matched for age, gender, and educational level; (2) study the influence of the main clinical variables (years of evolution of the disease, pharmacological treatment, and clinical service through which treatment is received) on the performance of patients on EEFF tasks; (3) explore the possible relationship between the severity of PS and the performance of patients on EEFF tasks; and finally (4) verify if the patients present clinically significant scores for any of the three frontal behavioral syndromes (Dorsolateral, Orbitofrontal, and Anterior Cingulate).

### Alterations in cool EEFF

As we expected, the patient group showed significantly poorer performance than the control group on the *cool* EEFF tasks.

Regarding *working memory*, our data agree with findings in the previous literature (Forbes et al., 2009; Menon et al., 2001). In our study, patients showed poor performance on the two components of WM that we evaluated: *coding/maintenance of information* (Sternberg-type task) and *updating of information in WM* (2-Back task). Accordingly, various studies have highlighted the importance of WM in PS, such as hallucinations, formal thought disorders, or delusions (Díaz-Caneja et al., 2019).

Regarding hallucinations, a relationship has been observed between auditory hallucinations and deficits in verbal WM tasks (Bruder et al., 2011). Given these findings, it has been argued that WM deficits could predict the presence of auditory verbal hallucinations(Jenkins et al., 2018); even from a first psychotic episode (Gisselgård et al., 2014), or in the general population who have more frequently experienced psychotic experiences (hallucinations and delusions) but who have not been diagnosed with mental illness (Rossi et al., 2016). In this sense, it has been observed (in a group of adolescents with reports of psychotic experiences in the absence of clinical disorder) that increasing the WM load when moving from a 2-back task to an overload in the 3-back task was associated more strongly with a higher level of psychotic experiences. Similarly, and through signal detection theory (SDT), an increase in false alarms was found to be associated with stronger psychotic experiences, as well as greater false recognition of auditory signals and words (Rankin & O’Carroll, 1995), suggesting that decreased discrimination is a characteristic of positive psychotic phenomena (Bentall & Slade, 1985; Rossi et al., 2016).

Deficits in WM have also been implicated in formal positive thinking disorders. According to authors such as Goldman-Rakic (Goldman-Rakic, 1994), the derailment, the loss of logical associations in thought, the inability to perceive causal relationships, or typical behavior through internal mental representations are the product of weaknesses in WM. Similarly, symptoms such as tangentiality, poor planning, cohesion of discourse, and deficiencies in information processing have specifically been linked to a dysfunction in updating and retrieving information from verbal WM (McGrath et al., 1997).

Regarding the performance on the task that assesses the capacity for *cognitive flexibility* (Number–Letter task), our patients only showed higher task-switching costs in reaction times (TSC^TR^) compared to controls, but not a higher cost of switching in terms of errors committed (TSC^Error^) (categorizing a stimulus as consonant or vowel, according to the position of the squares in which it appears, compared to the performance when they do not have to make such a change).

In patients with PS, although some studies have found that a poorer ability to change the mental set allowed for distinguishing patients who presented auditory verbal hallucinations from those who did not (Siddi et al., 2017), other studies have found no evidence of this relationship (Berman et al., 1997) reporting a preserved capacity for cognitive flexibility in schizophrenia (Greenzang et al., 2007; Hilti et al., 2010). In this sense, Meiran et al. (Meiran et al., 2000) have proposed that the deficits in cognitive flexibility found in patients with schizophrenia (evaluated using task-switching paradigms (Allport et al., 1994) could reflect a poorer memory for remembering information from the context of the task rather than a deficit in cognitive flexibility. In their study, although the patients had a higher TSC^TR^, they were as efficient as controls when executing the task. To test this hypothesis regarding the difficulty to remember the keys that indicate change and their corresponding response, the authors evaluated healthy participants in conditions in which the information about the meaning of the response had to be acquired again on each trial. It was found that these participants showed a task-switching cost pattern similar to that of patients, suggesting that in patients with schizophrenia there could be a difficulty in remembering the instruction that signals the change in task, rather than dysfunction in the TSC.

Regarding the *planning* task (Tower of Hanoi), our patients only differed from the control group in terms of latency in the short planning trials. Still, they did not make more errors than the controls, suggesting a preserved ability, albeit with slower processing speed. A possible explanation for these results could be found in studies suggesting that cognitive deficits in schizophrenia may be mediated in part by a reduced processing speed that interferes with cognitive performance rather than by cognitive failure itself (Mathias et al., 2017; Rodríguez-Sánchez et al., 2007).

### Alterations in hot EEFF

Regarding the most socio-emotional or *hot* EEFF, compared with the control group, the patients showed significantly poorer performance on two of the tasks studied: the *recognition of facial emotional expressions* and the task that evaluates the *theory of mind* (Hinting Task). These two processes—both the recognition of facial emotions and the recognition of intentions, emotions, and thoughts—are complementary processes that are necessary for adequate social functioning (Jáni & Kašpárek, 2018).

In our study, patients demonstrated a poor ability to identify and label facial emotions compared to controls; this was observed both for basic or innate facial expressions and those that are more complex. Therefore, our data suggest that patients with PS may present a marked deficit in identifying and categorizing emotions on the face. Although some studies have related these deficits more to negative symptoms than positive symptoms (Andrzejewska et al., 2017; Kohler et al., 2000), other studies have reported similar results. The latter found that in patients with PS, there was a generalized deficit in the perception of facial emotions, both in the earliest stages of the disease and in the more chronic stages, highlighting the possibility that this deterioration in the identification of emotions could represent a marker of trait susceptibility, rather than being a sequela of the disease (Barkl et al., 2014; Chan et al., 2010).

Mixed results can be found in the current scientific literature regarding the deficits that patients present in *theory of mind* (ToM). Some meta-analyses have found no clear affectation of ToM in patients with PS (Chan & Chen, 2011; Ventura et al., 2010), while other studies have found that patients show over mentalization in which an excessive and inaccurate attribution of mental state goes beyond the social cues provided (Abu-Akel, 1999; Fretland et al., 2015; Wastler & Lenzenweger, 2020). In a similar vein, the neurocognitive model developed by Frith (Frith, 2004) suggests that although patients with marked PS have an intact ToM in the sense of understanding that other people have mental states, they show poor performance due to difficulties in accurately monitoring and using contextual information, leading them to make incorrect inferences about the mental states of others. According to the model, these difficulties would lead to a breakdown in communication and eventually to a formal thought disorder and difficulties in distinguishing between subjectivity and objectivity, in addition to holding false beliefs in the form of delusional convictions. Our results, therefore, are in line with those studies that highlight ToM involvement in patients with a predominance of PS since, compared to the control group, our patients showed a significantly poorer ability to infer the true intention of indirect speech.

### Clinical variables and patient performance in hot and cool EEFF tasks

Regarding the clinical variables analyzed (years of evolution of the disease, clinical treatment device, and type of pharmacological treatment), we only found differences concerning the variable *years of disease evolution*. These differences were observed only in the planning task (Tower of Hanoi) of the *cool*, where patients with a short disease evolution (less than 11 years) made fewer errors in the short planning condition (less than five movements are required to complete the model) compared to the group with long disease evolution (more than 11 years). Subsequent analyzes with the control group revealed that patients with a short disease evolution showed similar performance to controls. This finding could suggest that, in patients with shorter disease evolution, the deficits in planning could be less severe or are more preserved in the earlier stages but deteriorates as the disease progresses, showing greater involvement.

### Positive symptoms and hot and cool EEFF

Regarding the relationship between PS and performance on EEFF tasks, the *Formal Thought Disorder* symptom showed a significant correlation with performance on both *cool* and *hot* executive functions tasks. Specifically, this symptom was positively correlated with cognitive flexibility and planning and negatively correlated with ToM. The *bizarre behavior* symptom was only positively correlated with working memory, and the delusional symptom was negatively correlated with planning.

These results highlight the importance of EEFF of a more cognitive or *cool* type in PS, particularly in WM. Although we also found correlations with cognitive flexibility, and with planning, in this sense, it is also interesting to note that the correlation with planning was observed in the reaction time condition, which could suggest that in these patients, there is a marked decrease in the processing speed that could interfere with performance on the task (Mathias et al., 2017).

Regarding the correlation found between *formal thought disorders* and ToM, our results are in line with the suggestions of authors such as Frith (Frith, 2004) and Corcoran (Corcoran, 2004), where formal thought disorders, such as the use of neologisms, excessive use of pronominal referents, rigid thinking, and idiosyncratic speech, arise from not considering the state of knowledge of other people. These patients, therefore, do not recognize the difference between their state of knowledge about a subject and the state of knowledge of the other person. This difficulty in separating the two states of knowledge would thus be manifest in a significant failure of ToM.

Finally, it is worth highlighting our findings from the perspective of the three-dimensional model described by Liddle et al. (Liddle & Morris, 1991) In this model, the PS of schizophrenia include two different factors, one related to the distortion of reality (*hallucination symptoms* and *delusions*), and a disorganizing factor (e.g., *formal thought disorder* and *bizarre behavior*). The disorganization symptoms are those that would present a stronger relationship with the neurocognitive deficits in comparison with distortion of reality symptoms (Cuesta & Peralta, 1995; Ventura et al., 2010). Similarly, in our study, disorganization symptoms were most strongly correlated with performance on both *cool* and *hot* executive EEFF tasks compared with distortion of reality symptoms. Therefore, these results could suggest that within the dimension of PS, there are two types of symptoms that differ in terms of cognitive functioning.

### Frontal behavioral syndromes and positive symptoms

Concerning the issue of whether the patients with PS present any of the three frontal behavioral syndromes, we found that a large percentage of our patients presented a clinically significant score on the three syndromes. A high score (> 65) in the subscales that make up the FrSBe test is a robust indicator of behavioral abnormalities related to the frontal system (Grace & Malloy, 2001). Therefore, as we expected, our results point to a possible affectation of the three fronto-subcortical circuits in this population. A higher percentage of the patient group appeared to suffer from *Dorsolateral syndrome* (81.5%) and *Anterior Cingulate syndrome* (77.8%), while 59.3% also presented high scores for *Orbitofrontal syndrome*. Similar results were reported by Ruiz-Castañeda et al. (Ruiz-Castañeda et al., 2020) in patients with schizophrenia with a predominance of negative symptoms (see Appendix 1). In this study, a high percentage of patients with a predominance of negative symptoms also presented clinically significant scores for the three syndromes, particularly Dorsolateral syndrome (72.20%) and Anterior Cingulate syndrome (69.70%), while a lower percentage indicated the presence of Orbitofrontal syndrome (33.30%). This could suggest that in schizophrenia, patients also have a wide variety of behavioral abnormalities related to the involvement of the fronto-subcortical circuits.

Dorsolateral syndrome is mainly characterized by the presentation of problems in EEFF. Our patients, therefore, showed a wide variety of behaviors resulting from this syndrome, such as the difficulty to anticipate future events; the inability to use strategies to retain information and put it to proper use; in addition to difficulties when performing more than one task at the same time. Our patients also showed difficulty in self-reflection and monitoring of their behavior along with an inability to adjust their behavior according to the feedback provided by other people.

Regarding Anterior Cingulate syndrome, our patients presented behaviors related to poor initiation, psychomotor retardation, persistence, loss of energy and interest, personal hygiene problems, and apathetic behaviors. Regarding the Orbitofrontal syndrome, a part of our sample reported an inability to inhibit actions or behaviors appropriately; these patients reported impulsive, hyperactive, and socially inappropriate behaviors, as well as a difficulty to modulate their emotional states, presenting poor emotional control including emotional lability or irritability.

## Implications and conclusions

The main findings of our study, following our proposed objectives, are described below. First, patients with a predominance of PS in schizophrenia presented specific deficits in *cool* and *hot* EEFF in comparison with healthy controls. The patients showed poorer performance on all the *cool* EEFF explored (WM, cognitive flexibility, and planning), with a larger effect size observed in WM. Regarding the *hot* EEFF, they showed worse performance in recognition of emotions and ToM. However, our patients did not show differences in the Iowa Gambling Task that assesses decision-making under conditions of uncertainty. Performance on this task has been consistently implicated in adequate functioning of the orbitofrontal area of ​​the brain. In this sense, it is interesting to note that compared to the Dorsolateral and Anterior Cingulate syndrome, a lower percentage of our patients showed clinically significant behaviors associated with Orbitofrontal syndrome; therefore, a possible explanation for our results could be the conservation of this brain area in our sample of patients.

Regarding the influence of *clinical variables*, patients with a short disease evolution showed better execution of planning than patients with a long evolution. No difference was observed in the execution of the tasks depending on the type of clinical device to which the patients belonged or the psychopharmacological treatment.

Regarding the relationships between PS and poor performance in executive functioning, it was the *formal thought disorder* symptom that showed a significant correlation with performance on both *cool* and *hot* EEFF tasks. Specifically, this symptom correlated with cognitive flexibility, planning, and ToM. The bizarre behavior symptom only correlated with working memory, while both *hallucinations* and *delusions* were related to planning.

Concerning the three frontal behavioral syndromes (Dorsolateral, Orbitofrontal, and Anterior Cingulate), we found that a high percentage of our patients presented all three syndromes, the most prevalent being Dorsolateral syndrome (81.5%), followed by Anterior Cingulate (77.8%), and Orbitofrontal syndrome (59.3%).

Finally, we consider that our findings make a significant contribution to the literature in several ways:

1. There is a scarcity of studies in the literature that explore EEFF in patients with schizophrenia distinguished according to the predominance of positive versus negative symptoms. This approach offers the advantage of analyzing more precisely the relationship between clinical symptoms and EEFF, avoiding the rigidity implied by a nosological classification of schizophrenic disorder.
2. A further contribution of this work comes from our attempt to explore in more depth the EEFF in patients with schizophrenia by analyzing both the *cool* and *hot* components. The advantage of adopting this perspective is that it allows us to take a finer approach to determining the neuropsychological involvement in the functions studied, which will inform the development of appropriate neuropsychological and psychotherapeutic interventions for this patient population.
3. Another noteworthy aspect of this study is the measurement instruments used. We have employed a battery of computerized neuropsychological tasks based on experimental paradigms developed within cognitive neuroscience. These evaluative instruments allow us to obtain valid and precise measurements of the patient's performance under study. They also allow the study to be replicated with other populations for comparison of results.
4. Finally, another important aspect to emphasize is the involvement of fronto-subcortical circuits in patients with PS. Studies of other populations have reported that these circuits are altered in, for example, patients with brain damage. However, to our knowledge, this is the first study to explore the links between behavioral abnormalities related to the frontal system and the PS of schizophrenia.

## Limitations

This study must be viewed in light of several limitations. First, a small sample was used, which could reduce the statistical power of our study. Second, the study was not carried out using the blind method because the recruitment and subsequent evaluation of the patients were carried out in the hospital context, so the evaluator knew the clinical characteristics of the participant. However, and to have greater control over the presentation of stimuli and the collection of responses and thus minimize the influence of evaluator biases, the study used an extensive battery of computerized neuropsychological tests to evaluate both *hot* and *cool* executive functions.

Finally, regarding the clinical variable of pharmacological treatment, the sample was not divided according to an estimate based on chlorpromazine equivalents. And although we found no differences in performance on the EEFF tasks according to the medication they were taking at the time of the evaluation ((1) medicated patients vs. patients without medication; (2) typical and atypical vs atypical/without medication), our results should be interpreted with caution, since some studies have highlighted the possible beneficial effects of atypical medications on general cognitive functioning (Buchanan et al., 1994; Meltzer & McGurk, 1999; Purdon et al., 2000). However, according to Harvey et al. (Harvey & Keefe, 2001), some of these studies used poor methodologies, and their results should be regarded as preliminary, requiring replication in further studies conducted with higher methodological standards.

## Figures and Tables

**Fig. 4:**
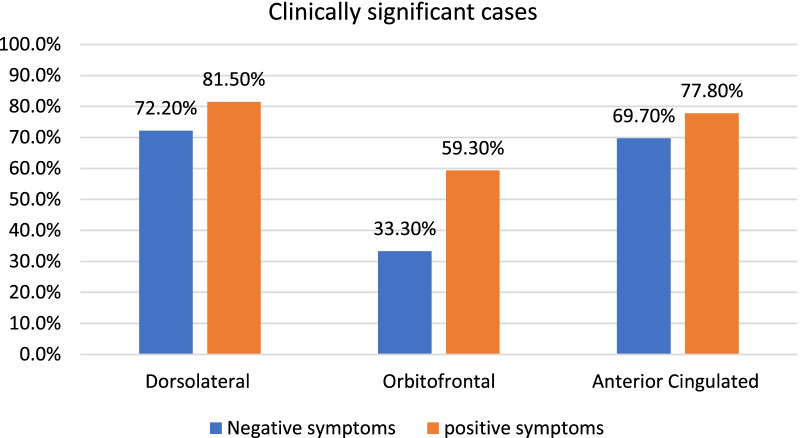
Percentage of clinically significant cases in fronto-subcortical syndromes in patients with positive and negative symptoms. The negative symptom scores have been adapted from the study of Ruiz-Castañeda et al. (2020)

## Data Availability

The datasets used and analyzed during the current study are available from the corresponding author on reasonable request.

## Appendix 1

See Fig. 4.
